# The Library Part of Our Journal

**Published:** 1848-10

**Authors:** 


*The Library Part of our Journal.*—We would invite the attention of our our readers to the Library part of the present volume of the Journal, in which we commence, with the present number, the publication of a translation, by P. H. Austen, M. D., of Baltimore, of a very excellent French work on the Surgical Diseases of the Mouth and its Adjoining Structures, by M. Jourdain. The design of the translator is to give to the dental and medical professions a complete treatise on these diseases, and to this end he proposes such corrections and additions to the original work, as the present advanced state of the science demands. For this task Dr. A. is eminently qualified. We would, therefore, commend the work to every dental practitioner in the United States, as one from the careful study of which he cannot fail to become better qualified for the discharge of the duties of his profession.

We have not the time just now, however much inclined, to discuss the urgent necessity which exists for the greater cultivation, in the dental profession, of a general medical and surgical education. We all see the low state of the profession at large, and we all regret it. But this state will continue, and these regrets will avail us little, so long as the responsible, and, when rightly understood, the noble duties of the dentist, are assumed by men possessed of no higher qualification than is found in the artisan's uneducated apprentice, and actuated by no more ennobling motive than stirs the breast of the sordid Californian gold digger; so long as *such* are countenanced and encouraged by the prominent among us, who *ought* to have more regard for the honor of a profession, in the reputation of which their own is involved, than to be guilty of so suicidal a policy.

Education, cultivation and refinement are indispensable in our profession; but it is *education* especially that we need, and to it we look for the elevation of the dental art. We hopefully anticipate the day, which we trust is not very distant, when the only door of admittance to our profession shall be through a thorough medico-dental education, and when dental science shall have an established literature. We therefore hail with pleasure every attempt towards this desirable result, and in this light, we view the work now claiming our attention.

In a recent conversation with Dr. Flagg, of Boston, he expressed to us a strong wish that we should publish, in the Library part of our Journal, a translation of the work of Jourdain, and we doubt not that many others will feel gratified at the present undertaking. The intimate and important connection of the structures whose diseases are here treated, with the teeth cannot fail to strike every reflecting mind. Without a thorough acquaintance therewith, no one is justified in regarding himself as qualified for the practice of dentistry.—*Balt. Ed.*

*Note by Translator.*—To the friends of M. Jourdain, the translator would offer an apology for the liberties which he has found it necessary to take with the original. The Introduction is presented entire, as also the first two chapters; but in the subsequent pages, it has been deemed necessary to omit personal and local discussions, which cannot possibly interest or profit the present day; to set aside confessedly obsolete and exploded ideas, and not unfrequently to reduce diffuseness of style and description to that concise accuracy which should characterize all surgical works. This course, in itself necessary to the interest and benefit of the translation, is the more demanded that the requisite additions, which will be as brief as is consistent with clearness, may not swell the work, already sufficiently large, to too formidable a size.

The additions of the American Editor are enclosed in the body of the work within brackets, [ ] and, when thrown into the form of foot notes, are marked by the signature *Tr.*

